# Automated monitoring of alcoholic fermentation: trends and challenges

**DOI:** 10.1007/s13197-025-06528-0

**Published:** 2026-01-16

**Authors:** Tomáš Horváth, Szilárd Kun, László Sipos, Duc Trung Pham

**Affiliations:** 1 School of Computing, Engineering and the Built Environment, Edinburgh Napier University, Sighthill Court, Edinburgh, EH11 4BN Scotland, UK; 2 Institute of Food Science and Technology, Hungarian University of Agriculture and Life Sciences, Villányi út 29-43, Budapest, 1118 Hungary; 3 Centre for Economic and Regional Studies (ELTE CERS), Eötvös Loránd University, Tóth Kálmán utca 4, Budapest, 1097 Hungary; 4 Institute of Academia-Industry Cooperation, Faculty of Informatics, Eötvös Loránd University, Pázmány Péter sétány 1/C, Budapest, 1117 Hungary; 5 Institute of Computer Science, Faculty of Science, Pavol Jozef Šafárik University, Jesenná 5, 040 01 Košice, Slovak Republic

**Keywords:** Alcoholic fermentation, Sensors, Electronic nose, Electronic tongue, Data analytics, Artificial intelligence

## Abstract

The progress of fermentation, an important step in spirit production, needs to be monitored regularly to detect possible faults. Automated monitoring of fermentation, however, is often limited to only a few parameters of the mash such as, and mainly, its temperature. With the advance of sensor technology and data analytics, various solutions to automated fermentation monitoring emerged, mainly for the beer and wine industry, however, these are not yet critically evaluated and compared. Thus, scientific articles on automated monitoring of alcoholic fermentation are reviewed and evaluated here according to the type of sensors used, the type of fermented material, and the reproducibility and feasibility of the presented solutions. Possible data analytics methods to utilize are introduced and their pros and cons are discussed. A critical evaluation from scientific and industrial perspectives is provided with prospects for the distilling industry where mashes of various states of matter, inhomogeneity and viscosity can appear. Key findings and conclusions of this review are: Electronic nose and electronic tongue biosensors are a promising direction in the area. A publicly available database on recorded data from e-nose and e-tongue as well as other sensors on fermentation monitoring is needed but still missing. Current solutions on automated fermentation monitoring are rather isolated studies, conducted in laboratories, yet to be evaluated and tested in industrial environments. The use of machine learning techniques in these studies, in general, does not comply with the well-established standards in data science and artificial intelligence.

## Introduction

*Controlled fermentation* (Jacques et al. 2003) is important in spirit production to ensure optimal conditions for the used yeast strain in the mash while blocking the development of unwanted microorganisms. The main parameters of the mash to monitor and control are its temperature, pH, dry matter and sugar content, level of $\hbox {CO}_2$ emission, volatile acid content, as well as physical, chemical and microbiological cleanness (Jacques et al. 2003).

**Fig. 1:**
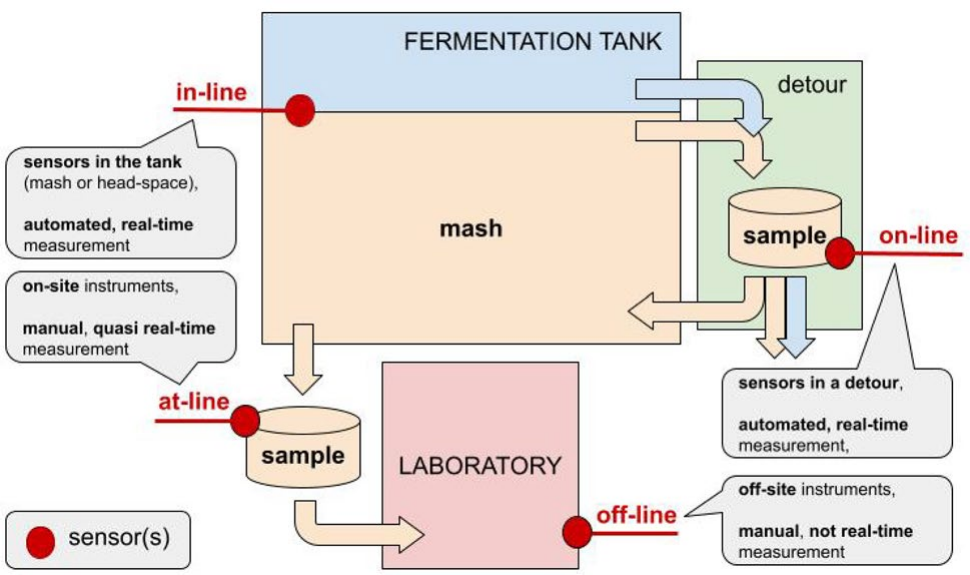
Four ways of monitoring fermentation (based on Cargalo et al. (2022))

Fermentation parameters can be monitored *in-line*, *on-line*, *at-line* or *off-line* (Fig. 1). The majority of distilleries have laboratories equipped with instruments of various complexity and precision, depending on their financial possibilities. However, human labor/expertise is needed to carry out the measurements which, regardless of the used equipment, are difficult to be fully automated. Thus, fermentation parameters are commonly monitored at-line or off-line.

Regarding the automation and automated monitoring of alcoholic fermentation, the vast majority of industrial solutions, however, focus only on the temperature parameter. There is a large variety of commercially available fermentation tanks with jacketed coolers measuring and controlling the temperature of the mash.

This survey, using the Google^®^ search engine^1^ (due to its efficiency and indexing ability of the databases of various publishers), focuses on *scientific publications*, which are being analyzed and evaluated according to the following factors:

- *Type of sensors*: This not only defines the type of data collected but also determines where the given measurements are performed, i.e. inside the mash (e.g. thermometer, e-Tongue), in the head-space of the tank (e.g. $\hbox {CO}_2$ sensor, e-Nose) or outside the tank (e.g. scale);
- *The fermented raw material*: This determines the state of matter of the mash, important when sensors are placed inside the mash. The homogeneity of the mash should also be considered here, especially when the fermentation tank is not equipped with a stirrer (e.g. the temperature of the core of the mash, in the middle of the tank, might be different from its temperature near the wall of the tank);
- *Reproducibility and feasibility*: An important attribute of a scientific work is its reproducibility and, preferably, the availability of the used materials and methods as well as the used or generated data. A related issue is the level of expert knowledge and equipment needed to implement the presented solutions.

## Materials and methods

### Fermentation parameters and related sensors

Various parameters to measure during fermentation and the related sensor types, found in the literature for measuring these parameters, together with their advantages and disadvantages, are listed in Table 1.

**Table 1:** Fermentation parameters and related sensor types used for their measurement

Parameter	Sensor	Comment / advantages ($\oplus$) / disadvantages ($\ominus$)
℃	Resistance temperature detector (RTD)$^1$	$\oplus$ accurate, $\oplus$ stable, $\ominus$ not cheap
	Thermistor, thermocouple, semiconductor	$\oplus$ cheap, $\oplus$ commonly used, $\ominus$ less accurate, $\ominus$ less stable
$\hbox {CO}_2$	Electrochemical gas sensors (Table 2)	$\oplus$ compact, $\oplus$ cost-effective, $\ominus$ limited lifespan, $\ominus$ affected by temperature, humidity, ethanol, $\ominus$ measures only the presence of $\hbox {CO}_2$
	Non-dispersive infrared (NDIR)	$\oplus$ accurate, $\oplus$ stable, $\oplus$ measure the amount of $\hbox {CO}_2$, $\ominus$ affected by water molecules and some gases (e.g. methane)
% alc.	Electrochemical gas sensors (Table 2)	$\oplus$ compact, $\oplus$ cost-effective, $\ominus$ limited lifespan, $\ominus$ affected by temperature, humidity
	IR spectroscopy sensors	$\oplus$ precise , $\oplus$ efficient, $\ominus$ accuracy depend on calibration
	Bio-electronic sensors	$\oplus$ relatively accurate, $\ominus$ short lifespan
pH	Glass electrode sensors	$\oplus$ inexpensive, $\ominus$ frequent calibration needed
	Optical pH sensors	$\ominus$ short lifetime, $\ominus$ narrow or rather higher ranges (above pH 5), more research needed for use in fermentation monitoring
Extract & sugar content	Bio-sensors	$\oplus$ specially tailored, $\ominus$ short lifespan
	$\hbox {iSpindle}^{2}$, Wifi digital $\hbox {hydrometer}^3$	$\oplus$ open-source, $\oplus$ open hardware, $\ominus$ not working (tested) in pulpy mashes and pomace (e.g. fruit brandy, grappa)
Aroma components	Electrochemical gas sensors (Table 2)	For volatile compounds, $\oplus$ compact, $\oplus$ cheap, $\ominus$ limited lifespan, $\ominus$ affected by temperature, humidity, ethanol, $\ominus$ lot of preliminary measurements/data needed for recognition, $\ominus$ sensor drift (Ye et al. 2021)
	e-Tongue	For non-volatile compounds, $\ominus$ not cheap, $\ominus$ limited lifespan, $\ominus$ affected by temperature $\ominus$ lot of preliminary measurements/data needed for recognition, $\ominus$ sensor drift (Ye et al. 2021), $\ominus$ memory effect (Kovacs et al. 2020)

$^{1}$https://atlas-scientific.com/blog/types-of-temperature-sensors/

$^{2}$https://github.com/universam1/iSpindel/blob/master/docs/README_en.md

$^{3}$https://hackaday.io/project/21386-wifi-digital-hydrometer

**Table 2:** Some MQ type gas sensors (https://robu.in/mq-series-gas-sensor/) with possible use in fermentation monitoring

Sensor	Sensed gas
MQ-3	Alcohol, ethanol, smoke
MQ-4	Methane, CNG gas
MQ-5	Natural gas, LPG
MQ-9	Carbon monoxide, flammable gasses
MQ-135	Carbon monoxide, ammonia, benzene, alcohol, smoke
MQ-136	Hydrogen sulfide gas
MQ-137	Ammonia
MQ-138	Benzene, toluene, alcohol, acetone, propane, formaldehyde gas, hydrogen
MQ-214	Methane, natural gas
MQ-303A	Alcohol. ethanol, smoke

The “MQ” designation refers to “Methane Quality”, which is a manufacturer-specific series name for these types of Metal Oxide Semiconductor (MOS) sensors

### Methods to analyze sensor data

The data generated by the majority of sensors introduced in the previous section can be represented as *time-series*^2^ of various complexities, such that *univariate*, when only one sensor is deployed, or *multivariate*, in case of using more sensors.

The goal of analyzing fermentation monitoring data is to extract correlations and patterns between the different parameters of fermentation and use these patterns to predict its further development. Since the gathered data is a standard data type, its preparation is a routine work assuming that the data analysis is performed by a specialist with sufficient knowledge. The evaluation and application of the obtained results (patterns and/or models), however, is subjective to their complexity as well as the user (scientist, brewer, etc.).

The literature is not uniform on systematization of data analysis methods, however, these are usually divided into two main groups, depending on the purpose of the analysis:

- *Data mining* (DM) (Moreira et al. 2018) refers to the group of *descriptive methods* applied to gain deeper insight into the data. Thus, it involves methods returning interpretable patterns, which are eventually translated into new domain knowledge.
- *Machine learning* (ML) (Alpaydin 2010) refers to the group of *predictive methods* resulting in *models* (mathematical functions), “trained” on recorded observations (labeled *train data* instances), used for predicting the outcomes (*labels*) of future observations (unlabeled *test data* instances). Here, to make better predictions, interpretability of models is usually traded for their prediction accuracy.

A possible categorization of various data analysis methods is introduced in Table 3.

The majority of DM and ML techniques have certain parameters, called *hyper-parameters*^3^ (HPs), which have to be carefully *tuned* for the resulting models to be optimal according to pre-selected *evaluation criteria* (e.g. high prediction accuracy).

**Table 3:** Our systematization of the main data analysis methods

Descriptive (data mining)	Statistical	Univariate	Numerical indicators
			Complex indicators
		Multivariate	
			Factor analysiss
	Algorithmic	Clustering	Crisp versus fuzzy
			Convex versus non-convex
		Pattern mining	Shapes and motifs
			Itemsets and rules
Predictive (machine learning)	Easy-to-interpret	Linear	Regression
			Classification
		Non-linear	k-nearest neighbors
			Bayesian networks
			Markov models
			Decision trees
			Random forests
	Hard-to-interpret		Adaboost
			Gradient boosting
			Support vector machines
			Neural networks

## e-Nose and e-Tongue in food and alcoholic beverage monitoring

The volatile components of a sample can be measured using *e-Nose*, an array of gas sensors, each of which detects certain (not necessarily only one specific) gas molecules in air. Combined measurements from these sensors generate unique response data, the so-called *fingerprint* of the sample. Subsequently, DM and ML methods are used to find specific patterns in these fingerprints that can be used for various purposes such as, for example, product authentication or fault detection. The aroma components of a sample, within the solution, can be analyzed using *e-Tongue*. e-Tongue works on the same principles as e-Nose, except that it is used for liquids instead of gases (Tan and Xu 2020).

E-nose and e-Tongue require proper calibration, i.e. correlating sensor responses to known concentration of certain compounds in the sample (Rudnitskaya 2018). Without calibration, only the presence of these compounds in the sample can be determined reliably but not its quantity.

Measurement performance characteristics (selectivity and specificity, limits of detection and quantification, resolution, range, accuracy, precision, sensitivity and linearity, stability and ruggedness, response time) of sensors used in e-Nose and e-Tongue, as usual in the case of any other sensors, are brand-specific and have to be acquired from the manufacturer.

According to Tan and Xu (2020), using e-Nose and e-Tongue in uncontrolled sampling is difficult since the amount of volatile components depends on temperature, humidity and pressure. Also, a large amount of labeled data is needed to train reliable ML models, thus, a freely available information repository is needed. Moreover, due to different types of sensors, sample preparation procedures, training data sets and ML methods used, research works are difficult to compare. They recommend collecting data with standardized sensors and mention the *matrix effect* when measurements in real environments differ from measurements in laboratories.

Wang and Chen (2024) draw attention to the selection of sensors suitable for a given task and that appropriate corrections for noise and *sensor drift*, when measurements are not consistent due to environmental changes (e.g. temperature, humidity) or the aging of sensors, are not utilized in most of the works. They question the application of Deep Neural Networks (DNNs) to small amount of data and claim for standardized data collection processes.

Karakaya et al. (2020) draw attention to *bio-electronic* noses (Lim and Park 2014) for spirits monitoring, the use of which are not yet spread.

Cheng et al. (2021) analyze the research and development of *compact e-Nose* applicable to several fermentation tanks of different types and sizes, common in craft breweries. They favor classical ML methods for their applicability in small computing devices connected into the *Internet of Things* (IoT).

Adeleke et al. (2023) provide a bibliometric review on the use of IoT for remote monitoring of fermentation, including solid-phase fermentation of baijiu. The review focuses mostly on the technologies used for inter-device communication and data analysis. They emphasize the importance of monitoring and forecasting the fermentation process to achieve effective quality and process control.

Ye et al. (2021), similarly to Liu et al. (2023) (see below), discuss the issues of data analysis with particular attention to sensor drift and noise. Ye et al. (2021) note that DNNs have achieved good results using *transfer learning*, however, due to the lack of freely available benchmark data, it is difficult to compare various results published in the literature.

Besides the findings similar to Ye et al. (2021), Liu et al. (2023) note that available data were collected under laboratory conditions, not taking into account the disturbing effects occurring in real conditions (e.g. foreign odors). They highlight the advantages of interpretable models which facilitate further research. Similarly to Wang and Chen (2024) and Cheng et al. (2021), they recommend the use of traditional ML methods.

Cargalo et al. (2022) discuss the importance of *soft sensors* that can be used to estimate hardly measurable parameters of the fermentation from its easily measurable parameters using mathematical or ML models (Zhu et al. 2020).

Baldwin et al. (2011) note that the presence of alcohol and $\hbox {CO}_2$ makes the application of e-Nose (and e-Tongue) difficult. They recommend combining these sensors with other sensors (e.g. *electronic eyes*).

Peris and Gilabert (2013) note that further research with much more samples and sensor types is required for industrial-scale spread of e-Nose and e-Tongue applications.

Santos et al. (2017) state that most applications of e-Nose are related to the examination of raw materials and differentiation of the finished products.

Varnamkhasti et al. (2011) analyze the interfering effect of ethanol and water on e-Nose. Ethanol can suppress the recognition of components present in lower concentrations what can be eliminated with appropriate calibration using reference samples. The disturbing effect of water can be eliminated by solid-phase micro-extraction, full automation of which is still costly.

Toko and Tahara (2016) discuss the use of lipid/polymer membranes developed for e-Tongue that can detect basic flavors.

Lozano et al. (2016) point out that, in addition to water and ethanol, the presence of sulfur in wine samples is a problem. They discuss works on detection of wine defects, such as acetic acid or defects caused by fungi.

Lozano et al. (2016) refer to two works which may be interesting for eliminating the difficulties caused by ethanol and water: In (Ragazzo-Sanchez et al. 2009) water and ethanol are separated with a gas chromatograph (GC) and only the other components are let into the e-Nose. In (Gil-Sánchez et al. 2011) a “wet e-Nose”^4^ is used to measure the acidity of the wine.

Rodríguez Méndez et al. (2016) recommend to combine e-Tongue with other sensors (Buratti et al. 2011; Legin et al. 2003). Branchini et al. (2016) praise the advantages of *sensor fusion*^5^ for more effective perception (Rodriguez-Mendez et al. 2004). Rodríguez-Méndez et al. (2016) argue for a holistic approach when e-Nose and e-Tongue are combined, and for a freely accessible database to facilitate further research and development. They recommend the widest possible use of bio-sensors, combined carefully since respective enzymes function under specific conditions.

Vasilescu et al. (2019) discuss the chemical background of biosensors with particular interest in determining glucose and phenol content of wines. They introduce their monitoring system for small and medium-sized wineries (costing approximately 15k Euros).

According to Seesaard and Wongchoosuk (2022) e-Nose is mainly used to determine the quality and authenticity of products. They state that recent research in e-Nose development is focusing on its size, sampling method and data processing algorithms (also vote for a public benchmark database). They suggest that future research should rather focus on developing novel and more sensitive gas sensors.

Buratti and Benedetti (2016) are monitoring changes in the parameters of the mash during fermentation using the fusion of e-Nose and e-Tongue and statistical models, however, note that more research is needed.

Violino et al. (2020) review low-cost and (possibly) open-source and open-hardware technologies. The benefits of these technologies, although promising, have not yet been scientifically proven due to a lack of sufficient literature. They also discuss the issue of traceability and transparency,^6^ achievable by *blockchain* technology.

**Table 4:** Survey articles on the applications of sensors in the food industry with the number of referenced articles related to alcoholic beverages and alcoholic fermentation

Survey	Sensor $\hbox {type}^{1}$			Number of references		
	eN	eT	O	Total	Alc. Beverages	Alc. Fermentation
Cargalo et al. (2022)			$+$	266	5	1
Adeleke et al. (2023)			$+$	52	12	5
Tan and Xu (2020)	$+$	$+$		106	5	–
Wang and Chen (2024)	$+$			368	8	–
Karakaya et al. (2020)	$+$			297	8	–
Baldwin et al. (2011)	$+$	$+$		110	13	–
Cheng et al. (2021)	$+$			210	13$^{2}$	–
Ye et al. (2021)	$+$			125	9$^{2}$	–
Liu et al. (2023)	$+$			297	1	–
Santos et al. (2017)	$+$			77	35	2
Varnamkhasti et al. (2011)	$+$			73	27	–
Rodríguez-Méndez et al. (2016)	$+$	$+$		105	73	4
Vasilescu et al. (2019)	$+$	$+$		120	67	12$^{4}$
Seesaard and Wongchoosuk (2022)	$+$			198	24	2
Buratti and Benedetti (2016)	$+$	$+$		45	17	12
Peris and Gilabert (2013)	$+$	$+$		42	5	1
Violino et al. (2020)			$+$	–	–	–

$^{1}$Abbreviations: eN = e-Nose, eT = e-Tongue, O = Other

$^{2}$Monitoring acetic acid in few of these articles

$^{3}$Sum of references in chapters related to alcoholic fermentation (overlaps)

$^{4}$6 papers are concerned with monitoring malolactic fermentaion

The discussed surveys are summarized in Table 4, indicating the types of sensors mentioned in them, the total number of articles referenced by them (column 5), the number of referenced articles related to alcoholic beverages (column 6) and the number of referenced articles related to alcoholic fermentation (column 7).^7^

According to Table 4, the literature of monitoring alcoholic beverages is relatively small, about 16% compared to all articles concerning food monitoring. These, however, can mostly be classified into two groups: The first group contains articles related to classification of alcoholic beverages into different groups based on aroma profiles, e.g. predicting the type of beer or establishing the authenticity of wine. The second group includes articles that measure the quality or some of the properties of alcoholic beverages, e.g. the sugar content of wine or the contamination of beer.

## In-line and on-line monitoring of alcoholic fermentation

According to Table 4, very little research deals specifically with monitoring of alcoholic fermentation. Moreover, a substantial part of these works are based on at-line or off-line measurements (Fig. 1) using laboratory equipments (e.g. GC or mass spectrometer) which cannot be easily integrated into an automated monitoring pipeline. The few works related to on-line and in-line monitoring of alcoholic fermentation are summarized in Table 5.

Albanese et al. (2011) introduce an instrument, based on flow injection analysis,^8^ using a horseradish-peroxidase based biosensor to measure the phenol content and a glucose oxidase-based biosensor to measure the sugar content of the must with good accuracy compared to GC. They also introduce a lactate oxidase-based biosensor to accurately measure the lactic acid content of the must, and an alcohol oxidase-based biosensor to accurately measure its alcohol content. The must is diluted to get linear responses from the developed e-Tongue.

Austin et al. (1996) first pervaporate the brewery wort with the help of teflon and silicone membranes and, after it is transformed to a gaseous state, measure the alcohol content using an electronic gas sensor.

Becker et al. (2001) measure the density of the fermenting wort in a 300 m^3^ fermentation tank using an ultrasound-emitting/receiving piezo crystal sensor mounted outside the tank, placed above the yeast sediment (the conical part of the tank), and calibrated based on wort temperature. The method is later improved by Hoche et al. (2016).

Buonocore et al. (2021) measure the weight of discharged $\hbox {CO}_2$ using an electronic scale placed under the tank (90 ls of wort loses approximately 3–3.5 kg of weight during fermentation).

The gases in the fermentation space of a small laboratory bioreactor are led into a GC using an automated pump by Calderon-Santoyo et al. (2010), using their patented *back-flush* method, where the alcohol content of a synthetic mash is measured. Volatile aroma compounds are separated from water and alcohol by the GC and then introduced into an e-Nose to compare the aroma-forming ability of two types of yeast inoculated into a synthetic medium.

Cañete-Carmona et al. (2020) monitor the amount of $\hbox {CO}_2$ produced during wine fermentation by an IR $\hbox {CO}_2$ sensor placed in a special tunnel.

Cavinato et al. (1990) use an external optical sensor to measure the alcohol content in fermentation tanks equipped with observation window. The light is guided from the light source to the tank and from the tank to the infrared spectrometer by optical cables, avoiding sampling which can lead to contamination of the mash.

de Andrade et al. (2021) collect data from a thermometer to control the home brewing process with Raspberry PI$^{12}$. They note that the compatibility, flexibility and reliability of this technology does not yet meet industrial expectations. However, it is suitable for craft and home breweries.

The amount of $\hbox {CO}_2$ in the atmosphere of the brewery is measured using Arduino^9^, $\hbox {CO}_2$ sensors and wireless technology by Hawchar et al. (2022). They draw the attention to the power supply of wireless technologies,^10^ needing regular battery replacement.

Lidén et al. (2000) train an Artificial Neural Network (ANN) on e-Nose data, labeled using liquid chromatograph (LC), and predict, relatively precisely, the glucose, the acetaldehyde and the ethyl acetate content of a synthetic medium, and also its glycerol and alcohol content, however, with less accuracy. They measure the $\hbox {CO}_2$ content in the gases discharged from the fermentation tank by an IR optical sensor. They note that ANNs are capable of finding complex non-linear relationships and to measure these components indirectly, however, more data need to be collected.

Lidgren et al. (2006) measure the glucose content of a synthetic medium with their SIRE^®^ technology in which the system dispenses enzymes to the sensing electrodes through a membrane. Filled with various enzymes, this sensor can be used flexibly.

Mamolar-Domenech et al. (2023) use a hydrophone, lowered into the (synthetic) mash, to measure the sounds emitted by the $\hbox {CO}_2$ bubbles correlating with their size (lower mash density = smaller bubble dimensions = higher frequency sounds) to monitor the progress of fermentation. The sound strongly correlates with the temperature of the mash, however, with a small delay caused by the heat absorption ability of the mash.

Pinheiro et al. (2002) measure the development of aroma substances in fermenting must for 20 days with a commercial e-Nose containing 32 gas sensors. Their experiment show that, essentially, the same “fingerprints” were obtained for the samples containing only ethanol and the samples containing the same amount of ethanol with different added flavoring substances (the humidity of samples was the same). Thus, aroma components present in very small quantities are hidden from the sensors by high amount of ethanol. Therefore, either the ethanol must be extracted from the samples, extracting also some aromatic substances, or the aroma substances must be concentrated. They recommend pervaporation of samples, e.g. using a silicone membrane.

Ranasinghe et al. (2013) emphasize the importance of placing thermometers into the fermentation tank, however, they don’t recommend fixed sensors as these can be damaged during cleaning. They note that large tanks, especially if not equipped with stirrers, require monitoring at multiple points due to temperature differences, often at 5 m depth, so the sensors must withstand the pressure. They introduce a 2.5 m long tubular probe with 7 thermometers placed at a distance of 30–50 cm from each other, which can be lifted out when cleaning the tank.

Sainz et al. (2013) use wireless technology to transmit accurate temperature data measured in the fermentation tank, utilizing free hardware^11^ and open source code, to the processing unit. They note that wireless technologies can be installed and relocated easily.

Shrake et al. (2014) introduce a LED colorimeter for measuring the color and phenol content of red wines, giving similar results to UV spectrophotometry. Yeast cells and $\hbox {CO}_2$ are filtered out from the samples using a 2 $\mu$m membrane filter.

Tamo and Hilario-Tacuri (2020) measure wort temperature with simple sensors controlled by a Raspberry PI^12^.

In the “smart barrel” system of Tomtsis et al. (2016) the color and purity of the wine during fermentation is measured by an LED RGB^13^ color sensor. The pressure of $\hbox {CO}_2$ in the fermentation space is measured by a pressure gauge. The number of bubbles passing through the airlock is detected by an optical sensor (photo-diode). They measure the temperature of the mash with a thermometer dipped into it, while another one measures the atmospheric temperature. They also use a digital pH meter immersed in the mash to measure its pH, and MQ type gas sensors (Table 2) placed in the head space of the tank to measure its alcohol content.

Uehara et al. (2019) measure the temperature of koji in different points of fermentation trays with a thermometer. In further steps of the production, the temperature of sake mash is measured at the bottom, in the middle and at the top of the fermentation tank (Uehara et al. 2018).

Vasilescu et al. (2019) use a (previously calibrated and corrected) carbon electrode to measure the phenol content of must, showing strong correlation with its color. They measure the glucose content using a glucose oxidase-based biosensor and the reducing sugar content via a gold electrode catalytic sensor, with relatively accurate results. Samples are automatically diluted according to the progress of the fermentation in the ratios from 1:1000 (at the beginning) to 1:50 (at the end), to achieve linear sensor responses. Sensors are changed every 5 days.

Vošahlík and Hart (2021) use a liquid-insulated thermometer and a Raspberry PI$^{12}$-compatible pH meter to measure the temperature and pH of rice wine mash.

Rice wine mash temperature is controlled in Zhang and Wang (2013) by continuously measuring the temperature of the fermentation room according to which a light bulb, used as a heat source, is switched on or off.

**Table 5:** Articles on in-line and on-line monitoring of alcoholic fermentation

Publication	Monitoring				$\hbox {R}^{2}$	$\hbox {F}^{3}$
	Medium	Measured property	Sensor $\hbox {type}^{1}$	Mode		
Albanese et al. (2011)	Wine	% alc., phenols, lactic acid, glucose	Bio-eT	In-line	3	3
de Andrade et al. (2021)	Beer	℃	Th	In-line	3	1
Austin et al. (1996)	Beer	% alc.	eT	On-line	3	1
Becker et al. (2001)	Beer	brix	Ultrasound	On-line	3	2
Buonocore et al. (2021)	Beer	$\hbox {CO}_2$	Scale	On-line	3	1
Calderon-Santoyo et al. (2010)	–	% alc., aroma	GC, eT	On-line	3	3
Cañete-Carmona et al. (2020)	Wine	$\hbox {CO}_2$	oS	On-line	3	1
Cavinato et al. (1990)	–	% alc.	oS	In-line	3	3
Lidén et al. (2000)	–	% alc., glucose, $\hbox {CO}_2$, ethyl acetate, acetaldehyde	eN, oS	On-line	3	3
Lidgren et al. (2006)	–	Glucose	Other	On-line	2	3
Mamolar-Domenech et al. (2023)	–	$\hbox {CO}_2$	Hidrofon	In-line	3	1
Pinheiro et al. (2002)	Wine	Aroma	eN	On-line	3	1
Ranasinghe et al. (2013)	Wine	℃	Th	In-line	3	2
Sainz et al. (2013)	Wine	℃	Th	In-line	3	1
Shrake et al. (2014)	Wine	Color, phenols	oS	In-line	3	3
Tamo and Hilario-Tacuri (2020)	Beer	℃	Th	On-line	3	1
Tomtsis et al. (2016)	Wine	℃, pH, color, $\hbox {CO}_2$, % alc.	Th, pH, eN, oS	On-line	2	2
Uehara et al. (2018)	Sake	℃	Th	In-line	3	1
Uehara et al. (2019)	Koji	℃	Th	In-line	3	1
Vasilescu et al. (2019)	Wine	Phenols, glucose	Bio-eN	On-line	3	3
Vošahlík and Hart (2021)	Rice wine	℃, pH	pH, Th	In-line	3	1
Zhang and Wang (2013)	Rice wine	℃	Th	On-line	3	1

$^1$Abbreviations: eT = e-Tongue, Th = Thermometer, GC = Gas Chromatograph, oS = Optical Sensor, eN = e-Nose, pH = pH meter

$^2$R = Reproducibility (3 levels): 1 = Missing description of method and experiments; 2 = Missing (incomplete) description of method or experiments; 3 = Reproducible research

$^3$F = Feasibility (3 levels): 1 = Can be made at home; 2 = Can be made in a well-equipped workshop; 3 = Can be made under laboratory conditions (e.g. biosensors or use of nanotechnologies Loira et al. (2020))

## Discussion

More than half of the articles on in-line and on-line monitoring of alcoholic fermentation (Table 5) is concerned with only one parameter of the fermentation, such as temperature, $\hbox {CO}_2$, dry matter or sugar content, or the alcohol content. These parameters are, however, not mutually independent and their changes show specific correlation patterns during the course of fermentation, regardless of the mash type, i.e. the alcohol content is increasing with increasing amount of $\hbox {CO}_2$ and increasing temperature (although, this one is usually controlled, thus, remain constant), while the dry matter or sugar content is decreasing.

### Industrial perspective

#### Investment payback

An important question for a distillery, as a business, is the return of investment. Based on a research carried out in small Australian wineries (Scrimgeour 2000), the installation price of industrial sensor technologies measuring the dry matter and sugar content of must is 22,500$ AUS with approximately 3 years return. The price of the monitoring system of Vasilescu et al. (2019) is also of a similar magnitude.

It is worth noting that wineries and breweries as well as distilleries of popular spirits (whiskey, vodka, rum, cachaça, tequila, cognac, etc.) can plan with greater certainty compared to distilleries of less popular spirits, such as fruit brandies where frost damages^14^ are recently causing substantial losses in fruit production due to changed weather conditions. Thus, the payback period mentioned above can be several times higher for small (craft) brandy distilleries.

Another issue is that biosensors, specifically made for monitoring alcoholic fermentation, can not yet be found on the market at reasonable prices.

#### Reliability

Inexpensive solutions, monitoring several fermentation parameters at the same time, could be used in craft distilleries. However, these have been tested only in laboratory environments.

Little information is available on the stability and lifetime of biosensors, which can range from short (approx. 1 week for the ethanol sensor) to long (several months for the glucose sensor). However, these solutions must be extensively tested in industrial environments in order to be able to safely determine which sensor, at what type of mash, and when needs to be replaced.

Regarding the reliability of e-Nose and e-Tongue, it is important to mention that extensive investigation and testing are needed to efficiently handle sensor drift and the matrix effect (Kovacs et al. 2020; Ye et al. 2021). The former could be handled by ML (Ye et al. 2021), while the latter is rather related to the issue of efficient cleaning of these sensors, especially e-Tongue (Kovacs et al. 2020), the automation of which (Olsson et al. 2006) needs more investigation for industrial settings.

Many solutions used Raspberry PI or Arduino technologies, not primarily developed for industrial environments, calling for further investigation to determine how fermentation environments (e.g. humidity, moisture, cleaning agents, etc.) affect their long-term operation. Industrial solutions tested by Scrimgeour (2000), however, were not reliable compared to reference methods^15^.

#### Ergonomics

Care must be taken to ensure that the use of monitoring solutions is safe from both physical and chemical point of view, to avoid accidents and contamination. The analyzed and discussed solutions have not been tested in this aspect.

To install most of the solutions, fermentation tanks must be modified, e.g. by installing a detour for the sampler (Fig. 1). Some of the modules of these solutions are located inside the tank, on the tank, or next to the tank, which can be damaged during cleaning.

Another question is the extent to which the mash is exposed to contamination during the replacement of the sensors. This is an important question, since the lifetime of some (bio)sensors is shorter than the fermentation time of some mashes.

At craft distilleries, fermentation tanks of several types and sizes are used, from small plastic barrels or steel tanks with floating lids, through few thousand liter tanks equipped with cooling jacket (the usual type), to tanks of several thousand liters capacity equipped with cooling jacket, stirrer and Cleaning-in-Place (CIP) system (mainly in larger businesses). Many articles lacked information on which types of containers the presented solutions could be used for, e.g. how the pressure in the tank, the pomace cap, or stirring of the mash affect the functioning of the solution.

### Research and development perspective

#### Reproducibility, feasibility and objectivity

As per Table 5, the presented solutions are clearly and precisely described in the respective papers, thus, can be easily reproduced and implemented given that the required expert knowledge and specialized equipments are available (e.g. chemical expertise, bio-receptors, transducers, electronics, fabrication equipment, reagents and solutions, laboratory tools for creating/assembling a biosensor).

Many articles note that there is no uniform and freely accessible database. However, there is no information in these articles about the availability of their data, despite the abundance of possibilities (e.g. public data repositories or, simply, linking a file on a website).

The presented methods were tested only in few mediums (types of wine, beer or some synthetic solution). Much more measurements are needed to get better insight into the presented solutions.

There are many small-scale and isolated studies that do not compare their work with other methods, despite that these are usually well documented. Thus, it is hard to compare two different methods developed to monitor the same property of the mash during fermentation.

#### Not liquid mashes

In case of liquid mashes of world’s most popular spirits, produced in largest quantities by large enterprises, such as whiskey, vodka, rum, cachaça, tequila, cognac, etc., monitoring technologies for wine and beer fermentation can be easily applied. However, the suitability of these technologies for mashes with state of matter, inhomogeneity or viscosity different from wine must or beer wort is not so straightforward and needs further research and investigation.

Examples of such mashes are fruit brandy mashes, i.e. fruit purees which can be relatively liquid (e.g. raspberries), pulpy (e.g. plums) or very dense (e.g. quince or pomace), causing some challenges for automated monitoring: First, the state of matter, inhomogeneity and viscosity can have a negative effect on the functioning of the membranes originally developed for filtering liquid mashes (Austin et al. 1996; Pinheiro et al. 2002; Shrake et al. 2014; Toko and Tahara 2016). Second, the heterogeneity of the various aroma substances of fruits as well as substances produced during fermentation (Fejzullahu et al. 2021), e.g. sulfur produced when fermenting sour cherry, can make the monitoring difficult or even mask the substances present in very small amounts.

The works concerning pulpy mashes are related to spirits made from rice, and are mostly monitoring temperature (except (Vošahlík and Hart 2021) where pH is also monitored). These spirits, such as sake or baijiu, are also very popular and produced in large quantities.

On the other hand, the tradition of making and consuming fruit spirits only extends to few countries, such as the “Schnaps” or “Obstwasser” known in German-speaking regions; “Pálinka” (Hungary), “Pálenka” (Slovakia and the Czech Republic), “Palincă” (Romania) in the Carpathians; “Raki” or “Rakija” in the Balkans. Moreover, fruit brandy production is mostly done by small family businesses and is seasonal in nature.

### Data analytics perspective

The methods used to analyze the data in the surveyed articles (Sect. 4) tend to two extremes of the wide palette of methods (Table 3): Either very simple, statistical, methods or methods that are difficult to interpret (PCA or ANNs) are used.

Several articles use ANNs (DNNs) to analyze data but, unfortunately, in most of the cases these models were not used or were not tested properly. Various data analytics issues, problems and challenges are discussed here using three articles (Lidén et al. 2000; Qi et al. 2017; Zhang et al. 2021) for illustration. We stress here that the purpose is not to criticize the choice of data analytics methods in these articles (they are not necessarily wrong), just to raise attention to the most important aspects to consider when analyzing fermentation monitoring data.

#### Training complex models on small data sets

It is important to choose ML models which are suitable for the given, usually small, data sets and not necessary force the use of popular models at all costs.

Qi et al. (2017) train a DNN on a data set of 30 samples taken from seven baijius. A similarly complex model is used by Zhang et al. (2021) on 6 samples taken from 10 baijius. In contrast to these, Lidén et al. (2000) use a simple ANN for 105 features extracted from time-series collected by 10 sensors on seven samples (i.e. do not use the ANN directly on the time-series), which seems to be a correct choice.

It should be noted here that small data sets can be “augmented” by generating synthetic data instances from the existing data set. However, in the case of data generated by sensors, especially if there are very small differences in the time-series related to different measurements, this might lead to inappropriately generated samples.

#### Missing or insufficient hyper-parameter tuning

Each ML method as well as the resulting optimized model have its specific HPs, that must be set before training, using different methods present in most ML software. Nevertheless, Qi et al. (2017) do not fine-tune at all, Zhang et al. (2021) only tune the HPs of the learning algorithm but not the architecture of the used ANN, while Lidén et al. (2000) perform both tuning of the HPs of the learning algorithm and the HPs related to the architecture of the used ANN, although, using a larger number of different HP settings during the tuning would probably lead to even better results.

#### Missing baseline models

In order to decide which ML model to choose for the given data, it is worth trying out several models, possibly of different paradigms (Table 3), and compare their performance using generally accepted, well-know statistical methods. Lidén et al. (2000) do not use any baseline method to compare their proposed model to, Qi et al. (2017) and Zhang et al. (2021) use some baselines but without fine-tuning their HPs, thus, the reported results might be biased.

#### Feature engineering

Raw data can contain information, also called features, that can be used to achieve good performance even with relatively simple ML models. The extraction of these information from the data is called feature engineering^16^ which, unfortunately, is not often utilized. Qi et al. (2017) and Zhang et al. (2021) do not perform feature engineering, while Lidén et al. (2000) do, resulting in a very good performance even with a simple ML model.

#### Green computing

Computers are responsible for approximately 5-10% of the world’s energy consumption, a significant part of which is used by artificial intelligence methods, including ML. Recently, an initiative called *green computing* has started to gain attention, advocating for sustainable computing practices.

In ML interpretation, this means that, if possible, one should use ML models that require little computing power to train and use, opting for compromise between the complexity and accuracy of the used ML methods and models. One of the ways to achieve this is by conducting feature engineering and HP tuning while using simple models (as Lidén et al. (2000) do), and use complex models only when it is, given the data and the task at hand, necessary. Simple models can run on simple hardware, which would be reflected in the price of the given solution as well.

#### Interpretability

The goal of analyzing fermentation data (in addition to accurate predictions or anomaly detection) should be to uncover hidden information and patterns in the data.

This can best be achieved by using models that can be easily interpreted by humans (Table 3). Unfortunately, we did not find any examples of using such models in the reviewed articles.

#### Benchmark data

As mentioned in many articles, there is a need for a freely available database that contains not only the raw sensor data (in the form of time-series) but, also, the data about the monitoring conditions (time, type of mash, type of sensors, etc.), called *meta-data*.

Since fermentation data and the related meta-data may be sensitive to the given company, data encryption and anonymization should be ensured and, if possible, privacy-preserving ML methods should be employed.

Although sensors can easily produce large amounts of data, we have to be aware of its reliable labeling, based on laboratory measurements or sensory evaluation by humans. The labeling method should also be recorded in the benchmark data.

A critical problem is that in many cases the collected training data might be imbalanced, meaning that one of the labels is very underrepresented in the data (e.g. the presence of acrolein in the mash is very rare under the use of proper mashing practices). These problems and their possible treatment, e.g. by using imbalanced ML techniques, must be taken into account during data collection, as well as analysis.

## Conclusions

Research on fermentation monitoring was reviewed in this article. Very few articles are focusing particularly on on-line or in-line monitoring of alcoholic fermentation. Moreover, these are rather isolated studies conducted in laboratories, yet to be evaluated and tested in industrial environments.

E-nose and e-tongue is a promising research direction, especially the development of novel biosensors for monitoring fermentation parameters. However, despite the large amount of well-reproducible technical articles, there is a lack, and need, of publicly accessible databases and uniform standards, necessary to draw conclusive results. Finally, the use of machine learning techniques in these studies, in general, does not comply with the well-established standards in data science and artificial intelligence.

There is a long-term research and development work yet to be conducted which should involve several subject areas including, but not limited to, brewing, wine making and distillation, sensor development, data engineering and artificial intelligence. We hope that this survey provides useful information for researchers in these fields focusing on automated monitoring of alcoholic fermentation, and contributes to the scientific knowledge on automation of the spirit industry.

## Data Availability

No new data or material was used, created or analyzed in this study. Data sharing is not applicable to this article.

## Footnotes

1 https://www.google.com

2 A series of values of a variable measured at given intervals is called time-series.

3 The name “hyper-parameter” (HP) is to distinguish it from a “parameter” of the resulting, optimized (mathematical) model.

4 The electrodes are placed in a wet textile which absorbs the volatile components.

5 Combining complementary information from different sensors, imitating human perception, e.g. of perceiving drinks first with eyes, then with nose and mouth.

6 The concept of “farm-to-glass”, slowly spreading in the alcohol industry, is following the well-known “farm to table” concept from the AgriFood industry.

7 E.g. in the first line, Cargalo et al. (2022) cites a total of 266 articles, of which 5 are related to monitoring alcoholic beverages, and 1 to monitoring alcoholic fermentation.

8 The sample is injected into a carrier solution with reagents before entering the detector.

9 https://www.arduino.cc.

10 By principles of “green computing”, the importance of technologies with low energy and computing requirements is increasing.

11 https://www.ti.com.

12 https://www.raspberrypi.com.

13 RGB is an additive color model, utilized in light-based digital devices, defining a broad spectrum of colors by combining varying intensities of red (R), green (G), and blue (B) light.

14 https://frostdefend.eu/en/

15 e.g.https://www.oiv.int/standards/compendium-of-international-methods-of-analysis-for-spirituous-beverages-and-alcohols.

16 PCA is also a kind of feature engineering, popular in the food industry.

## Code availability

No new code was generated in this study. Code availability is not applicable in this article.
